# Simultaneous radical cystectomy and nephroureterectomy: A case series

**DOI:** 10.1016/j.sipas.2025.100276

**Published:** 2025-02-25

**Authors:** Gavin G. Calpin, Steven M. Anderson, Mark Broe, Ijaz Cheema, Niall F. Davis, Dilly Little

**Affiliations:** a Department of Urology, Beaumont Hospital, Dublin 9, Ireland; b Royal College of Surgeons in Ireland, 123 St Stephens Green Dublin 2, Ireland

**Keywords:** Radical cystectomy, Nephroureterectomy, Urothelial cancer, Upper urinary tract, Outcomes

## Abstract

**Introduction:**

Simultaneous radical cystectomy and nephroureterectomy (RCNU) is a complex procedure. Although performed infrequently, RCNU may be indicated in certain cases of multifocal high grade urothelial carcinoma (UC) or muscle-invasive bladder cancer (MIBC) with an obstructed and atrophic kidney. The aim of this study was to review the indications, operative approach and outcomes for patients undergoing RCNU in our institution.

**Methods:**

A single-centre, retrospective review was performed. Cases were identified by reviewing theatre logbooks. Chart reviews were conducted and clinicopathological outcomes were recorded and analysed.

**Results:**

Eight patients were identified between 2015–2024. All were male and had a mean age of 66.4 ± 4.7 years. All patients underwent RCNU with ileal conduit formation. The surgical approach for the nephroureterectomy was laparoscopic in four cases and open in the remaining four. The mean post-operative length of stay was 11.6 ± 1.75 days. All patients had high grade UC, seven patients had MIBC at presentation. Only two patients received neoadjuvant chemotherapy, one of whom achieved a complete pathological response. In total, 62.5% (n=5) had T3/4 disease while 50% (n=4) had node positive disease. Two patients had synchronous upper tract urothelial carcinoma (UTUC) on final histology. The remaining cases had chronically obstructed and atrophic kidneys. Incidental primary prostate cancer was found in 62.5% (n=5). The mean follow-up was 31.6 ± 7 months, during which time there were four recurrences with three patients dying from metastatic disease. The mean overall survival was 21.8 ± 11.8 months and the mean disease-free survival was 19.3 ± 12.3 months.

**Conclusion:**

The results from this study demonstrate that combined laparoscopic and open RCNU is an effective treatment for both panurothelial cancer and MIBC with severe upper tract dysfunction.

## Introduction

Bladder cancer is the tenth most commonly diagnosed cancer worldwide accounting for 3% of cancer diagnoses every year[1]. Multifocal recurrences are characteristic of urothelial carcinoma (UC)[2] and can be challenging to manage, often requiring radical treatment. In rare cases, patients may have simultaneous bladder and upper tract tumours necessitating a radical cystectomy and nephroureterectomy (RCNU). This is a complex procedure and subjects patients to a significant risk of morbidity and mortality[3].

While bladder cancer is common, the outcomes of RCNU have been comparatively sparsely reported in the literature[4,5]. Furthermore, despite the growing interest in the use of minimally invasive techniques in the management of both upper tract urothelial cancer (UTUC) and MIBC, there is limited evidence analysing the optimal surgical approach in performing RCNU.

The aim of this study was to retrospectively review all patients who underwent a RCNU at our institution analysing the indications for surgery, the operative approach used as well as their peri-operative and longer term oncological outcomes.

## Methods

Approval for this retrospective study was obtained from the Clinical Research and Audit Department in Beaumont Hospital (CA2024/106). Cases were identified by reviewing theatre logbooks. Data from all patients who underwent a RCNU between 2015 and 2024 were collected by conducting chart reviews and obtaining pre-operative records, operative notes, post-operative records, histopathological reports, radiological imaging and clinic letters.

Detailed data regarding patient demographics and clinicopathological information were extracted for evaluation. This included indication for surgery, operative details, post-operative outcomes, histopathological diagnoses, oncological treatments, and survival outcomes. Operative details included operative approach employed for both the nephroureterectomy and radical cystectomy, diversion used, operative time, and estimated blood loss (EBL). Post-operative course included ICU/HDU admission, complications, blood transfusion, and length of stay (LOS). Histopathological information included primary diagnosis, the presence of malignancy in the kidney, ureter, bladder, and prostate, T and N stage, grade, presence of carcinoma in situ (CIS), and residual disease. Oncological information and outcomes included the use of neoadjuvant/adjuvant chemotherapy, recurrence, disease free survival (DFS), and overall survival (OS). Chart reviews were completed in June 2024.

## Results

Eight patients were identified who underwent their surgery between 2015 and 2024. All patients were male with a mean age of 66.4 ± 4.7 years, at the time of surgery. Two patients (25%) had neoadjuvant chemotherapy (NACT). Six patients (75%) underwent RCNU for their primary disease. Interestingly, 6 patients (75%) had bladder tumours involving a ureteric orifice (UO) resulting in a chronically obstructed and atrophic ipsilateral kidney. Three patients (37.5%) had malignancies with the clinical suggestion of multifocal tumours involving both the bladder and upper tract urothelium. Baseline patient characteristics are outlined in Table 1.

**Table 1:** Patient characteristics and indication for RCNU.

Patient	Gender	Age (years)	NACT	Primary Disease	Indication for RCNU
1	Male	72.9	No	Yes	T3 bladder tumour, 16% function in right kidney
2	Male	60.3	Yes	Yes	MIBC obstructing right kidney, 9% function
3	Male	71.4	No	Yes	T2 MIBC, obstructed left ureter and possible upper tract tumour, previous perforation
4	Male	58.2	No	No	Biopsies suspicious for HG malignancy in left renal pelvis and lower pole
5	Male	55.1	Yes	Yes	Previous pT2 at least MIBC, obstructed left kidney, 13% function
6	Male	70.0	No	No	Obstructing left kidney, 11% function
7	Male	74.1	No	Yes	Obstructive uropathy, 20% function on right
8	Male	69.2	No	Yes	Multifocal disease in bladder and upper tract

All patients underwent open radical cystectomies with ileal conduits acting as the urinary diversion in all cases. Regarding the operative approach for nephroureterectomy, a subcostal incision was used in two cases (25%) and a midline laparotomy approach was employed in two cases (25%), while the kidney and ureter were fully mobilised laparoscopically in the remaining four cases, three of which were hand-assisted laparoscopic (HAL) and one was fully laparoscopic. The mean operative time was 343 ± 33.8 min and mean EBL was 783 ± 450 mL. Operative details are outlined in Table 2.

**Table 2:** Operative details.

Patient	Nephroureterectomy	Cystectomy	Diversion	OT (mins)	EBL (mL)
1	Open (subcostal incision)	Open	IC	-	317
2	Open (subcostal incision)	Open	IC	-	1000
3	Laparoscopic (hand-assisted)	Open	IC	345	-
4	Laparoscopic (hand-assisted)	Open	IC	340	200
5	Laparoscopic (hand-assisted)	Open	IC	345	400
6	Open (midline laparotomy)	Open	IC	275	-
7	Open (midline laparotomy)	Open	IC	-	-
8	Laparoscopic	Open	IC	410	2000

The majority of patients had a relatively uneventful post-operative course with a mean LOS of 11.6 ± 1.75 days. Two patients (25%) suffered minor (Clavien-Dindo 1–2) post-operative complications, while two patients (25%) suffered more significant complications and were transferred to the intensive care unit after surgery. One of these patients, who underwent a laparoscopic nephroureterectomy and lower midline laparotomy for the cystectomy, developed type two respiratory failure and was subsequently discharged on post-operative (POD) day 16. The other patient, who underwent an open RCNU via a midline laparotomy, also had a respiratory complication, atelectasis, and was discharged on POD 16 but was readmitted on POD 28 with a wound dehiscence. The post-operative complications are summarised in Table 3.

**Table 3:** Post-operative course.

Patient	ICU/HDU	Complications	Blood transfusion	LOS (days)
1	No	None	No	9
2	No	None	No	10
3	No	Delirium	No	9
4	No	None	No	9
5	No	Ileus day 5	No	11
6	No	None	No	13
7	Yes	Atelectasis, wound dehiscence, readmission day 28	No	16
8	Yes	Pulomonary oedema, type 2 respiratory failure, transferred back to ICU	Yes	16

Histopathology demonstrated bladder malignancy in 7 patients (87.5%) with the other patient having a complete pathological response to his NACT (original histology showed pT2 high grade urothelial carcinoma). There was nodal involvement in 4 cases (50%) with 3 of these patients having metastases diagnosed within 1 month of RCNU. Two patients (25%) had multifocal urothelial malignancy involving the bladder and upper urinary tract (one involving the renal pelvis and one involving just the ureter). Five patients (62.5%) had synchronous prostatic malignancy detected; 2 were grade group 2 (Gleason 3+4) and 3 were grade group 1 Gleason (3+3). One patient (12.5%) had a positive margin in his prostatic urethra. Interestingly, this was the same patient who had multifocal disease in his bladder, kidney, and ureter. Table 4 shows histopathological details.

**Table 4:** Histopathological outcomes.

Patient	Bladder				Kidney			Ureter				Prostate		Residual Disease
	T	N	Grade	CIS	T	Grade	CIS	Location	T	Grade	CIS	T	Grade	
1	3b	1	High	Yes	-	-	-	-	-	-	-	-	-	No
2	0	0	-	-	-	-	-	-	-	-	-	2a	3+3	No
3	4a	0	High	Yes	-	-	-	-	-	-	-	-	-	No
4	is	0	High	Yes	is	High	Yes	Multifocal	is	High	Yes	2c	3+4	Yes (prostatic urethra)
5	4a	0	High	Yes	-	-	-	-	-	-	-	2c	3+4	No
6	3b	2	High	Yes	-	-	-	-	-	-	-	1a	3+3	Yes (mets)
7	4a	1	High	No	-	-	-	-	-	-	-	1a	3+3	No
8	1	2	High	No	3	High	No	-	-	-	-	TCC	Low	Yes (mets)

The mean DFS was 19.3 ± 12.3 months and mean OS was 21.8 ± 11.8 months. In terms of follow-up, 2 patients (25%) underwent adjuvant chemotherapy. Four patients (50%) developed a recurrence of their malignancy but three of these had metastatic disease detected within 1 month of their surgery. Of note, the mean length of time between the most recent pre-operative CT imaging and surgery in these three patients was 75 days compared to 35.8 days in the other 5 patients. The mean OS for these three patients was six months. The other patient developed a recurrence in the contralateral ureter after 23 months necessitating a radical nephroureterectomy, rendering them anephric and requiring haemodialysis, and was still alive with no other recurrence or metastases at the most recent follow-up 15 months later. This is the same patient who had multifocal tumours and residual disease in his prostatic urethra on histopathology. The four patients who had no recurrence have a mean DFS of 30 ± 7 months at most recent follow-up. Long-term and oncological outcomes are outlined in Table 5.

**Table 5:** Follow-up and oncological outcome.

Patient	Adjuvant CT	Recurrence	Death	DFS	OS
1	Yes	No	No	24 months	24 months*
2	No	No	No	26 months	26 months*
3	Yes	No	No	49 months	49 months*
4	No	Yes (right ureter)	No	23 months (underwent right RNU)	38 months*
5	No	No	No	21 months	21 months*
6	No	Yes (metastases)	Yes	1 month	4 months
7	No	Yes (metastases)	Yes	-	10 months
8	No	Yes (metastases)	Yes	0 months	4 months

⁎ denotes most recent follow-up

## Discussion

The purpose of this study was to review the clinical indications for and the peri-operative morbidity of RCNU in the management of complex cases of urothelial carcinoma. One interesting finding was how few patients had UTUC on final histopathology (25%). The question of how best to manage an atrophic and obstructed kidney in the setting of MIBC can be challenging. It is often not possible to adequately assess the urothelium of these kidneys and ureters to outrule a synchronous UTUC as the ureteric orifice is often not accessible to facilitate retrograde investigations such a retrograde pyelography or ureterorenoscopy. Furthermore, there is infrequently sufficient contrast excretion from the obstructed kidney for radiological investigations such as computed tomography urograms to be diagnostic. In these cases of diagnostic uncertainty, RCNU is a viable diagnostic and therapeutic option. This is supported by the results of this study as patients without multifocal tumours who underwent RNCU for MIBC with an obstructed and atrophic kidney obtained the best outcomes. None of these suffered significant post-operative complications, with just one case of delirium and one case of ileus seen, compared to the more significant complications seen in those patients with multifocal tumours. The mean LOS was 9.75 days and none of these patients have developed a recurrence of their cancer to date with an average follow-up duration of 31.6 months.

Unsurprisingly, the patients with multifocal disease on final histology had worse oncological outcomes than those without upper tract involvement. However, while one of the two patients who had multifocal disease died from metastatic disease just four months post-operatively, the other patient remains disease-free at most recent follow-up 38 months post-RCNU, despite needing a contralateral radical nephroureterectomy for disease recurrence at 23 months post-RCNU. This highlights that RCNU remains a suitable option for appropriately selected and counselled patients with multifocal urothelial cancer.

MIBC is a time-critical condition, and this is apparent in our patient cohort. Three patients had poor oncological outcomes with a mean OS of just six months. This is despite prompt surgical intervention and negative surgical margins following RCNU, and likely represents patients with pre-operative microscopic metastatic disease. An important consideration when counselling patients for RCNU is that they are often not suitable for NACT[3]. This is notable in our cohort, as only two patients had adequate renal function to receive platinum based NACT. While this is a common finding amongst patients with panurothelial cancer, recent developments in the use of neoadjuvant immunotherapy for both MIBC and UTUC may lead to a change in practice meaning more patients requiring RCNU may be suitable to receive neoadjuvant treatment in the future.

A recent systematic review analysed all available literature published on concurrent RCNU[3]. Interestingly, 70.3% of patients had multifocal disease involving both the upper and lower urinary tracts. Outcomes were otherwise similar to ours. The most common post-operative complication was paralytic ileus, 28% received a blood transfusion and the mean LOS was 13 days. They concluded that RCNU is a treatment option for concurrent upper and lower tract disease but that it should be cautiously utilised due to its associated morbidity and mortality risks. They also remarked that patient selection along with thorough discussion of risks and benefits of the surgery along with exploring all available treatment options with the patient remain the most important considerations in patients with complex urothelial carcinoma.

Another discussion point includes the incidental finding of prostate cancer in 62.5% (5/8) specimens. This rate is higher to that reported by Alsinnawi et al. who reported a 32.5% rate of prostate cancer in 110 radical cystectomy specimens[6]. However, their rate of clinically significant prostate cancer (Gleason grade group 2–5) in their series was 28.5% which is similar to our rate of 25.0%. It is an unsurprising finding that men of this age may have the incidental finding of low grade prostate cancer. Moreover, they all have a smoking history which is a known risk factor for prostate malignancy[7].

Whilst the results from this study and the literature to date support the use of RCNU in the management of MIBC with either concurrent UTUC or obstructed and atrophic kidneys, the optimum surgical approach remains unclear, with a reported comparable split between fully open, laparoscopic and hybrid techniques. No study in the literature has reported on outcomes based on the operative approach employed and therefore Zein et al. were unable to analyse this[3]. Where technically feasible, the authors have a preference for a hybrid approach, in which the nephroureterectomy is performed HAL, and the cystectomy and urinary diversion is performed open through the same incision. In this approach, a lower midline incision is used to access the peritoneum and a hand-assisted port (GelPort® Applied Medical Resources Corporation, Rancho Santa Margarita, California, USA) is then inserted and used to establish pneumoperitoneum. Once the renal hilum has been divided and the kidney and ureter have been maximally mobilised, the laparoscopic ports are closed, the hand-assisted port is covered using an Ioban dressing and the patients is repositioned (supine) and draped. The cystectomy is then completed through the initial lower midline incision, thereby avoiding the additional morbidity associated with a large midline incision that is often needed to complete a fully open RCNU. Whilst this is the author's preferred approach, it is not always feasible due to patient and tumour factors, as evident in our series. The authors also acknowledge that the recent advances in robotic-assisted laparoscopic surgery also provide an alternative method of reducing the morbidity associated with open surgery.

The main limitation to our study is the small sample size. As there were only 8 patients, we were unable to run univariate and multivariate analyses and therefore definitive conclusions on oncological outcomes and the optimum surgical approach cannot be derived. Its retrospective nature means it is inherently prone to bias. Moreover, not all data was obtained leading to an incomplete dataset. For example, operative time and EBL were not documented for every patient. However, the strength of the study is that we have obtained insight to the safety and efficacy of RCNU in select cases along with the intricacies.

In conclusion, RCNU can be considered as a viable treatment option in select complex cases of urothelial carcinoma. Careful and judicious patient selection is paramount and all treatment options should be explored. Patients without UTUC who have bladder tumours causing severe unilateral obstructive uropathy likely have the best immediate post-operative as well as longterm outcomes, though a sustained oncological response can also be seen in patients with multifocal disease.

## Funding statement

This research received no external funding.

## CRediT authorship contribution statement

**Gavin G. Calpin:** Writing – review & editing, Writing – original draft, Methodology, Investigation, Formal analysis, Data curation. **Steven M. Anderson:** Writing – review & editing, Writing – original draft, Methodology, Formal analysis, Data curation, Conceptualization. **Mark Broe:** Writing – review & editing, Supervision, Conceptualization. **Ijaz Cheema:** Writing – review & editing, Supervision, Conceptualization. **Niall F. Davis:** Writing – review & editing, Validation, Supervision, Methodology, Conceptualization. **Dilly Little:** Writing – review & editing, Writing – original draft, Visualization, Validation, Supervision, Methodology, Conceptualization.

## Declaration of competing interest

The authors declare that they have no known competing financial interests or personal relationships that could have appeared to influence the work reported in this paper.
